# Silage quality and biogas production from *Spartina pectinata* L. fermented with a novel xylan-degrading strain of *Lactobacillus buchneri* M B/00077

**DOI:** 10.1038/s41598-021-92686-y

**Published:** 2021-06-23

**Authors:** Marta Kupryś-Caruk, Renata Choińska, Agnieszka Dekowska, Katarzyna Piasecka-Jóźwiak

**Affiliations:** 1 Department of Fermentation Technology, Prof. W. Dąbrowski Institute of Agricultural and Food Biotechnology - State Research Institute, 36 Rakowiecka, 02-532 Warsaw, Poland; 2 Department of Microbiology, Prof. W. Dąbrowski Institute of Agricultural and Food Biotechnology – State Research Institute, 36 Rakowiecka, 02-532 Warsaw, Poland

**Keywords:** Microbiology, Environmental sciences

## Abstract

The aim of the current study was to determine the ability of the *Lactobacillus buchneri* M B/00077 strain to degrade xylan, its impact on the quality of silage made from the lignocellulosic biomass of *Spartina pectinata* L., as well as the efficiency of biogas production. In the model in vitro conditions the *L. buchneri* M B/00077 strain was able to grow in a medium using xylan as the sole source of carbon, and xylanolytic activity was detected in the post-culture medium. In the *L. buchneri* M B/00077 genome, genes encoding endo-1,4-xylanase and β-xylosidase were identified. The silages prepared using *L. buchneri* M B/00077 were characterized by a higher concentration of acetic and propionic acids compared to the controls or the silages prepared with the addition of commercial xylanase. The addition of bacteria increased the efficiency of biogas production. From the silages treated with *L. buchneri* M B/00077, 10% and 20% more biogas was obtained than from the controls and the silages treated with commercial xylanase, respectively. The results of the current study indicated the strain *L. buchneri* M B/00077 as being a promising candidate for further application in the field of pretreatment of lignocellulosic biomass.

## Introduction

Lignocellulose biomass such as perennial grasses, including species characterized by a C4 photosynthetic pathway e.g. *Spartina pectinata* L., as well as various agricultural residues and other herbaceous plants, could become an important feedstock for the production of biofuels^1^. Lignocellulose biomass is a commonly available and renewable energy source, and 55–75% of it consists of polymers (cellulose, hemicellulose, lignin) by dry weight and can be used for ethanol, as well as for biogas production during the anaerobic digestion (AD) process^2^. However, the main drawback of the lignocellulosic biomass is its structural heterogeneity and complexity causing the native biomass resistance to enzymatic hydrolysis creating thereby an obstacle for the AD process and finally, limiting biogas production. Hemicelluloses, especially xylan, if coated with crystalline cellulose restricts access to the cellulose microfibrils, making them water insoluble and recalcitrant to biodegradation^2,3^. Moreover, a high content of barely degradable fibre fractions in biomass intended for the production of gaseous biofuels limits the yield of methane produced^4^.

Many earlier studies have dealt with pre-treatment of lignocellulosic biomass to increase hydrolysis of cell wall structures. For this purpose, numerous physical, chemical and biological approaches have been proposed^5–9^. The efficient conversion of hemicelluloses, being an important class of plant cell wall polysaccharides, demands the common action of various enzymes because hemicelluloses, unlike cellulose, are not chemically homogenous^6^. Among the hemicelluloses, endo-1,4-xylanase and β-xylosidase play a major role in the depolymerization of xylan, the most abundant component of hemicellulose constituting approx. 20–30% of the herbaceous plant’s biomass^10^. The removal of xylan, except for facilitating the hydrolysis of cellulose; xylo-oligomers can also facilitate a decrease in enzyme inhibition and this leads to fewer inhibitors in the fermentation process^11^. The use of enzymes on an industrial scale is however, largely limited due to their high cost and limited availability^12,13^. Therefore, in recent years methods of hemicellulose bioconversion using microorganisms have been developed, because hemicelluloses, especially xylan, have many practical applications in various agro-industrial processes, including the production of lactic acid or biofuels^10,12^.

The biological pretreatment of lignocellulose biomass using microorganisms has been mostly associated with the action of fungi owing to their enzymatic capabilities^14–16^. However, microorganisms having xylanolytic activity are also found among various bacteria^2,3^. For instance Zerva et al.^17^ isolated waste xylanolytic microbial strains from orange-juice processing, among which *Pseudomonas psychrotolerans* and *P*. *oryzihabitans* showed the highest extracellular endo-1,4-β-xylanase activity with 280 U/mg protein. In a study by Guo et al.^2^, the strain *Pseudomonas boreopolis* G22 was used for pretreatment of wheat straw. This strain secreted abundant amounts of xylanase with an activity of 2.67–1.75 U/ml. Rumen microorganisms are considered to be unique source of enzymes with huge potential in the pretreatment of lignocellulosic biomass. But the application of rumen microorganisms in biorefinery technologies is limited due to the fact that many rumen microorganisms are difficult to culture and require unique growing conditions^18^. In this context, the use of lactic acid bacteria, a well-known group of microorganisms having wide application in the agro-food industry, has been found as a promising strategy. Lactic acid bacteria are commonly used in the biological pretreatment of biomass before producing biofuels^1^. Ensiling, as the traditional method used for preserving biomass, has a reported impact on cellulose conversion through enzymatic hydrolysis, which had led to improved saccharification for the production of bioethanol^19,20^. Many studies have showed that the ensiling process has a positive impact on enhancing the production of biogas^21,22^. A huge advantage of lactic acid bacteria is their diverse metabolic capacity which enables them to adapt to various environmental conditions^23^. It has been proved that LAB living in a plant environment utilize substrates derived from plant cell walls due to expressing enzymes involved in e.g. the breakdown of hemicellulose^24^. Nserko et al.^25^ reported *L. buchneri* strains capable of partial decomposition of arabinoxylans by releasing ferulic acid from arabinose side-chains owing to ferulic acid esterase activity. An impact of silage additives on biogas production from silages prepared using various types of biomasses was reported by Herrmann et al.^26^ and Zhao et al.^27^. Zhao et al.^22^ used the *Lactobacillus brevis* species and the xylanase enzyme for ensiling switchgrass. The additives promoted the decomposition of the cellulose and hemicellulose, as a results of which more organic acids were accumulated in the ensiled biomass leading to the rapid production of methane. However, it has been reported that *Lactobacillus brevis* is unable to degrade xylan since no xylanase gene is present in its genome^28^. Another study revealed that an obligate heterofermentative species—*Lactobacillus buchneri* CD034—which is closely related to *L. brevis,* has the ability to degrade xylan owing to several genes encoding endo-1,4-β-xylanase, β-xylosidase, and α-l-arabinofuranosidase^24^. The species *Lactobacillus buchneri* is the desired silage additive due to its specific properties: the ability to transform lactic acid into acetic acid and 1,2-propanediol. Acetic acid can inhibit the growth of moulds and yeasts and 1,2 propanediol can be further metabolized into 1-propanol and propionic acid, the latter of which also has antifungal properties that can also improve the aerobic stability of silages^29^. It has been demonstrated by Filya^30^ that the studied strain of *L. buchneri* used for ensiling biomass improved the aerobic stability of the silages obtained after their exposure to air. Heinl et al.^24^ claimed that *L. buchneri* CD034 has a unique ability to adapt to complex plant polymers derived from plant cell walls owing to the expressing enzymes involved in the degradation of arabinan, xylan, and glucan.

Taking into account the abovementioned consideration, the objective of the current work was to investigate the ability of a new *Lactobacillus buchneri* M B/00077 strain to degrade xylan during fermentation of lignocellulosic biomass of cordgrass (*Spartina pectinata* L.), its impact on overall silage quality as well as on the specific methane yield and biogas production. For this purpose, the metabolic capability of *Lactobacillus buchneri* M B/00077 was investigated in model in vitro conditions, and the activity of xylanolytic extracellular enzymes released by the bacteria into the medium was evaluated. Additionally, to assess the effect of used *Lactobacillus buchneri* M B/00077 on the hemicellulose content and biogas yield comparative tests with a commercial enzyme (xylanase) were carried out.

## Results

### Metabolic activity of ***Lactobacillus buchneri*** M B/00077

The metabolic capability of *Lactobacillus buchneri* M B/00077 to ferment different sugars was investigated in model in vitro conditions. The strain tested showed an ability to grow in medium with xylan as the sole carbon source (Table 1). However, its growth in the medium with xylan was an order of magnitude lower than the growth in the medium with glucose. In the post-fermented medium with glucose, the pH was much lower than in the medium with xylan or xylose and the lactic acid concentration was more than nine times higher than the concentration of acetic acid. Growth in the mediums with xylose or xylan as the sole carbon source resulted in a much lower concentration of lactic acid and a significantly higher concentration of acetic acid than in the medium with glucose. Additionally, the concentration of acetic acid in the mediums with xylose or xylan, was 59 and 81% higher than that in the medium with glucose (Table 1). Moreover, in the mediums with xylose or xylan the concentration of acetic acid was 87% and 96% higher compared to that of lactic acid in these mediums, respectively.

**Table 1:** Parameters determined in the medium after *Lactobacillus buchneri* M B/00077 incubation depending on the carbon source.

Carbon source	Organic acids (g/L)		The number of bacteria (log CFU/ml)	pH
	Lactic	Acetic		
Glucose	24.8 ± 1.25^a^	2.7 ± 0.12^b^	9.56 ± 0.01^a^	3.5 ± 0.10^b^
Xylose	2.3 ± 0.08^b^	4.3 ± 0.50^a^	8.50 ± 0.05^b^	6.0 ± 0.21^a^
Xylan	2.5 ± 0.11^b^	4.9 ± 0.38^a^	8.56 ± 0.05^b^	6.4 ± 0.18^a^

^a,b^Mean values (in rows) marked with different letters differ significantly, α = 0.05.

**Figure 1:**
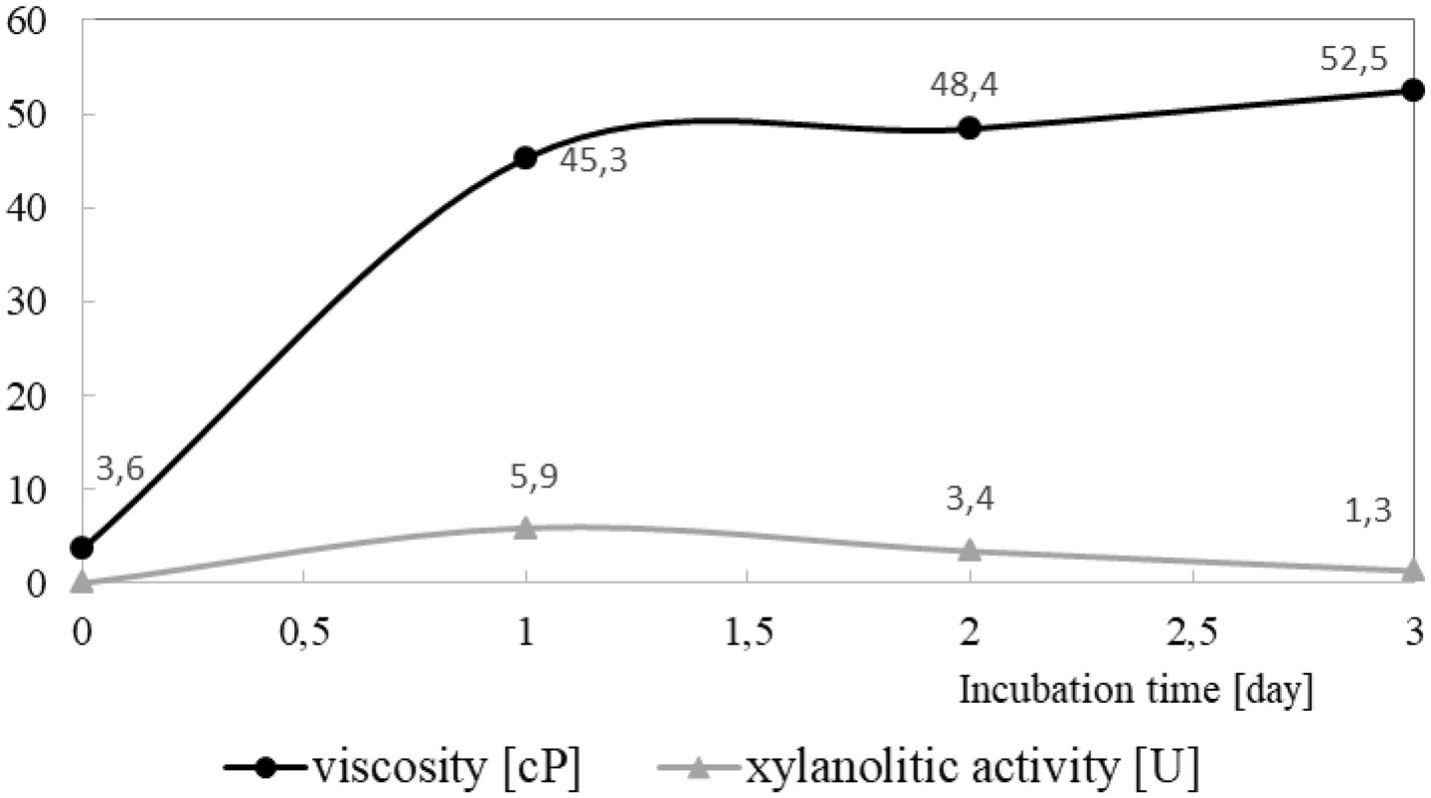
Viscosity of the medium with xylan as a sole carbon source during Lactobacillus buchneri M B/00077 incubation and xylanolytic activity determined in the post-fermented medium.

The activity of xylanolytic extracellular enzymes released by the bacteria into the medium was evaluated. The highest xylanolytic activity (5.9 U) was detected after 24 h of bacteria incubation, which was probably related to the activity of bacteria in the batch experiment. The decomposition of xylan and releasing of mono sugars resulted in an increase in the viscosity of the culture liquid. The highest increase in viscosity was detected during the first 24 h of bacteria incubation (Fig. 1).

### Identification of endo-1,4-β-xylanase and β-xylosidase genes of *Lactobacillus buchneri* M B/00077

The whole genome of the *Lactobacillus buchneri* CD034 strain isolated from stable grass silage was studied by Heinl et al.^24^. Based on this research, the presence of genes encoding enzymes: endo-1,4-xylanase (EC 3.2.1.8.) and β-xylosidase (EC 3.2.1.37), was determined. The products of PCR reaction in agarose gel are given in Supplementary Figure S1.

Two PCR products were obtained: a fragment of approximately 850 bp was obtained with xylanase primers and 650 pb with xylosidase primers. The DNA sequences obtained were examined using the Basic Local Alignment Search Tool (BLAST). The sequence producing the most significant alignments, for both query sequences, was from the *Lactobacillus buchneri* NRRL B-30929 strain deposited in GenBank under accession number CP002652.1 with 100% query coverage for the endo-xylanase sequence, 99.68% for the xylosidase sequence, 99.88% for the endo-xylanase sequence, and 99.68% for the xylosidase sequence. The second most similar sequence was from the *Lactobacillus buchneri* CD034 strain deposited in GenBank under accession number CP003043 with 100% query coverage for both sequences, 94.44% identity for the endo-xylanase sequence and 96.96% for the xylosidase sequence. All these results showed 0.0 expect value.

### Quality of silages and efficiency of biogas production

The impact of the *Lactobacillus buchneri* M B/00077 strain added to biomass on the chemical composition during ensiling was verified. The impact of the method used for preparing silages (with or without silage additives) on the composition of *Spartina* biomass is presented in Tables 2 and 3.

**Table 2:** Chemical composition of fresh and ensiled *Spartina pectinata* L.

Material	DM (%)	DDM (%)	ODM (% DM)	Crude protein (% DM)	Cellulose (% DM)	Hemi-cellulose (% DM)	WSC (% DM)	Crude fat (% DM)
Fresh grass	35.0^a^	54.2^a^	95.3^a^	4.8^a^	38.4^a^	34.8^a^	2.2^a^	1.25^a^
Control silage	34.6^a^	54.2^a^	94.8^b^	4.5^a^	37.9^a^	34.6^a^	0.65^c^	1.21^a^
Enzyme silage	34.1^a^	54.3^a^	94.8^b^	4.6^a^	38.0^a^	32.5^a^	0.77^b^	1.19^a^
LAB silage	34.8^a^	54.8^a^	94.8^b^	4.5^a^	37.8^a^	32.0^a^	0.65^c^	1.20^a^
SEM	0.42	0.16	0.06	0.04	0.17	0.51	0.16	0.01

*LAB* *Lactobacillus buchneri* M B/00077, *SEM *standard error of the mean.

^a,b^Mean values (in columns) marked with different letters differ significantly, α = 0.05.

**Table 3:** Organic acids in the silages and production of biogas from *Spartina pectinata* L.

Silage	pH	Organic acids (% DM)				Biogas production (dm^3^/kg_ODM_)		Efficiency of biogas production (%)
						Theoretical (max)	Observed	
		Lactic	Acetic	Propionic	Butyric	a	b	(b * 100)/a
Control	4.7^a^	1.20^a^	0.35^b^	nd	0.35^a^	684.7	592.7^b^	86.6^b^
Enzyme	4.4^c^	2.47^a^	0.34^b^	nd	0.12^a^	669.3	533.8^c^	79.7^c^
LAB	4.6^b^	0.85^b^	1.07^a^	0.87	0.12^a^	654.5	653.0^a^	99.8^a^
SEM	0.05	0.24	0.12	–	0.04	–	17.4	–

*LAB* *Lactobacillus buchneri* M B/00077, *SEM* standard error of the mean.

^a–c^Mean values (in columns) marked with different letters differ significantly, α = 0.05.

There were no significant differences in cellulose and hemicellulose content between the silages and fresh biomass (after harvesting, before ensiling). The hemicellulose content in the silages prepared with *L. buchneri* M B/00077 was 32.0% DM, whereas in the control silages 34.8% DM. It can be noticed that the effect of commercial xylanase and LAB on the hemicellulose content was comparable. The highest content of water soluble carbohydrates (WSC) was determined in the silages prepared with the addition of enzymes. The WSC concentration in the control and silages prepared using *L. buchneri* M B/00077 was at the same level (Table 2).

Ensiling had an impact on the organoleptic properties of the silages obtained (Table 3). Silages prepared using additives were characterized by an intensive acetic and wine odour, while the odour of the control silages was typical of biomass and had a predominant lactic acid odour. Growth of white mould was observed on the upper layer of the control silages. The pH of the silages obtained was lower than 5.0. Silages prepared with the addition of commercial xylanase had the lowest pH, whereas the highest was detected in the control silages.

The composition of organic acids varied among silages. The highest concentration of lactic acid was detected in those prepared with the addition of enzymes, and the lowest in the silages prepared using *L. buchneri* M B/00077. However, the LAB inoculated silages characterized the highest concentration of acetic acid that was almost three times higher than in the other silages. Propionic acid was detected only in the silages prepared with the addition of LAB. Silages prepared with *L. buchneri* M B/00077 were characterized not only by the highest concentration of acetic acid but also the highest efficiency in the production of biogas (Table 3). The concentration of methane in the biogas obtained amounted to 56.2% on average, irrespective of the type of silage (data not shown).

A significantly higher amount of biogas was obtained from silages treated with *Lactobacillus buchneri* M B/00077 and the amount of biogas obtained constituted almost 100% of the maximum theoretical production. Biogas production of 13.4 and 20.3% lower than theoretically calculated was obtained from the control and enzymatically treated silages respectively, while the lowest production was obtained from the silages prepared with the addition of commercial xylanase (Table 3).

## Discussion

The ability to degrade xylan is not a common feature of all known species of lactic acid bacteria. Boguta et al.^31^ examined 296 strains of *Lactobacillus* and genus closely related to *Pediococcus* and found only 56 strains able to utilize xylose. The ability to ferment C5 sugars is a feature of heterofermentative LAB, which utilizes those sugars through the pentosophosphate pathway. This process leads to the production of acetic acid^32^. In this process, xylose is first isomerized by xylose isomerase (XylA) to xylulose, which is further phosphorylated to xylulose-5-phosphate (X5P) by xylulokinase (XylB). Through the phosphoketolase pathway (PK pathway), X5P is finally converted into lactic acid and acetic acid^33^. The conversion of pentose results in lactate and acetate, with no CO_2_ production^32^. Acetic acid is a relatively weak acid, therefore its concentration has no impact on the pH of medium^34^. Therefore, in this study the biodegradation of xylan using the heterofermentative *Lactobacillus buchneri* M B/00077 strain did not cause high acidification of the post-cultured medium. According to Heinl et al.^24^, the metabolic activity of lactic acid bacteria is closely associated with the substrates available in their natural environment. One of the approaches in the selection of microorganisms for biotechnological application is the substrate-oriented strategy^31^ which focuses on the capacity of microorganisms to utilize a certain feedstock in order to select the best suited strain. The use of a strain isolated from the material, into which this strain is further introduced in the form of a preparation, seems to be the right approach in the above mentioned strategy.

In the current study *Lactobacillus buchneri* M B/00077 was able to grow in medium with xylan (and xylose) as the sole carbon source and synthesized higher amounts of acetate despite the heterofermentation process leading to equimolar amounts of lactate and acetate production^23^. *Lactobacillus buchneri* is known for its ability to metabolize lactic acid into acetic acid and 1,2-propanediol, when there is no glucose in the environment. This conversion occurs at low pH (3.2–4.3)^29^. Other authors have found that lactate metabolism is strain-dependent so there is a variation in utilization of lactic acid among the *Lactobacillus buchneri* species^35^.

A valuable finding of this study was that the *Lactobacillus buchneri* M B/00077 strain has genes encoding two critical enzymes required for the degradation of xylan: endo-β-1,4-xylanase and β-xylosidase^6,36^. This feature is strain specific and not all species of lactic acid bacteria produce the two enzymes. For instance, Chapla et al.^37^ investigated the xylanolytic activity of selected strains of *L. acidophilus and L. fermentum* genus and found that the strains studied indicated a lack of β-xylosidase activity. When grown in a medium with xylooligosaccharides as the sole carbon source, *L. acidophilus* and *L. fermentum* indicated the highest endo-xylanase activity (0.34 and 0.24 U/ml respectively) after 24 h of incubation. In another study it was found that *Leuconostoc lactis* SHO-47 and *Leuconostoc lactis* SHO-54 were unable to utilize xylan, probably because of the lack of a gene encoding endo-xylanase. The xylosidase activity of both strains was induced by xylose and a combination of xylobiose and xylotriose, and the activity of xylosidase was not found in the supernatant of the cultures but in the cell-free extracts^38^. In the study by Erlandson et al.^39^, genetic and biochemical evidence was found for a defective xylan degradation pathway in three LAB strains belonging to *Lactococcus lactis* although two of them were isolated from the plant environment.

In the current study a significantly smaller amount of biogas was obtained from silages prepared with the addition of commercial xylanase than from the control and LAB silages, although the silages treated with enzyme were characterized by a higher sugar and lactic acid content and increased rate of biogas production, particularly at the start of fermentation. The higher concentration of sugars and lactic acid indicated an increased intensity of the biochemical processes, probably due to the increased bioavailability of lignocellulosic structures for lactic acid bacteria naturally occurring in the plant biomass. The second phenomenon to be observed may be caused by inhibitors, i.e. compounds which interfere with the AD process and limit the production of biogas. Under experimental conditions short-chain fatty acids (C2–C6) formed in the process of transforming biochemical organic components, could be responsible for inhibiting anaerobic digestion. Their accumulation could have exceeded the upper maximum level enabling their use by methanogenic bacteria which, in consequence, could have been the reason for the production of less biogas from silages prepared with the addition of enzymes.

The highest amount of biogas was obtained from silages treated with *L. buchneri* M. The increase in the production of biogas in silages treated with *L. buchneri* M may be related to the increase in the acetic acid content, which is a product of the heterofermentative fermentation of pentoses released from hemicellulose (xylan) of plant material. These observations correspond to the results of other authors^40^. The use of efficient silage additives to enhance the production of biogas has been the subject of many studies in recent years some of which have proved that biological silage additives consisting of lactic acid bacteria strains showed positive effects on methane yields from biogas crops such as maize, sorghum, forage rye, triticale and switchgrass^26,27,40^. Other authors have claimed that this effect could be attributed to the increases in C_2_ to C_6_ volatile fatty acids and alcohols during ensiling, as these compounds, especially acetic acid, are direct precursors of methane production^40^. In the study by Herrmann et al.^26^, a regression model including acetic, butyric acid and ethanol accounted for 75–96% of the variation in methane yields. However, a high concentration of butyric acid in silages is not desirable and is considered to be a sign of the proteolytic activity of *Clostridium* bacteria, which finally leads to deterioration of the silage^41^. In the study by Ambye-Jensen et al.^1^ the effect of increasing acid concentrations in silages on the subsequent enzymatic hydrolysis of cellulose was observed. The authors assumed that synthesized organic acids were responsible for acid hydrolysis of the lignocellulose complex of grass biomass. The effect of increasing cellulose convertibility was more dependent on the content of the dry grass matter than on lactic acid bacteria used as a silage additives. Nevertheless, the study confirmed ensiling to be a useful biological pretreatment method for the conversion of green biomass. Vervaeren et al*.*^40^ reported that more divergent biological additives containing yeast or enzymes would be preferable to enhance the subsequent anaerobic digestion of silages than additives consisting of only homo- or heterofermentative LAB strains. Zhao et al.^27^ recommended the combined application of hemicellulose and *Lactobacillus plantarum* to improve the quality of silage and biogas production from rice straw. Others have reported the minor effect of lactic acid bacteria used as silage additives on the production of biogas from maize, suggesting that LAB do not have the ability to degradation of plant cell wall polysaccharides^42,43^. Furthermore, it was suggested that unlike LAB living in a dairy environment, the strains isolated from silages utilize substrates derived from plant cell walls^24^. Some authors claimed that the impact of the ensiling process on biogas yields should be carefully studied with particular emphasis on dry matter loss which occurs during ensiling^44^. This statement was supported by another study which showed that taking storage losses into consideration, that microbiological silage additives showed little effect on the methane production from various biogas crops^26^. Finally, some authors suggested that ensiling could increase the production of biogas even taking into account storage losses due to the more beneficial impact of the increase in biochemical accessibility as a result of the ensiling process^45^.

Another valuable observation made in this study was the occurrence of propionic acid which was detected only in silages prepared using *Lactobacillus buchneri* M B/00077. The lack of this acid in the control silages and silages prepared with the use of xylanase indicated the impact of *L. buchneri* M B/00077 on propionic acid production. Propionic acid is a natural inhibitor of mould growth and its presence is highly desired in silages, contributing to the increased aerobic stability of the ensiled material^41^. *Lactobacillus buchneri* species are characterized by their unique metabolic pathways^35^. It was reported that *L. buchneri* A KKP 2017/p synthesized propionic acid from 1,2-propanediol in the presence of cobalamin^46^.

## Conclusions

The genes coding key enzymes in xylan degradation i.e. endo-1,4-xylanase and β-xylosidase have been identified in the *Lactobacillus buchneri* M B/00077. Applying this strain as a silage additive for the lignocellulosic biomass of *Spartina pectinata* L. preservation had an impact on the composition of the silages and resulted in increased production of biogas. Thus, the results of the current research highlight the potential application of *Lactobacillus buchneri* M B/00077 for lignocellulosic biomass pretreatment promoting the economic conversion of biomass into biofuels.

## Materials and methods

### Microorganisms

*Lactobacillus buchneri* M B/00077 was isolated from spontaneously fermented grass of *Spartina pectinata* L. The identification of the strain was performed by analysis of the 16S rRNA sequence. The sequence obtained was compared with the sequences available in GenBank database. The strain was deposited in the Polish Collection of Microorganisms located at the Institute of Immunology and Experimental Therapy in Wrocław (Poland), under the number B/00077.

### Experiments in model conditions

#### Media growth test for *Lactobacillus buchneri* M

The metabolic activity of the *Lactobacillus buchneri* M strain was examined in standard MRS medium (containing 2% glucose) and in modified MRS medium, in which glucose was replaced by xylan from beech wood (SERVA, Germany) or D(+)-xylose (POCH, Poland). 100 ml of a particular medium (initial pH = 6.6) was inoculated with 1 ml of 24-h culture (inoculum) prepared in standard MRS medium (the number of bacteria in the inoculum was log 9.0 CFU m/l). Stationary cultures were incubated in flasks for 5 days at 30 °C. After a defined period of time the number of bacteria was determined using the ISO 15214 spread plate method and the pH of the medium was measured using the potentiometric method. The cultures were then centrifuged (10,000*g*, 10 min.) and supernatants were collected in which organic acids were detected (using the HPLC method).

### Determining the xylanolytic activity of the *Lactobacillus buchneri* M B/00077 strain

The activity of extracellular enzymes released into the medium by the tested strain was measured. For this purpose *Lactobacillus buchneri* M B/00077 was grown in liquid MRS medium with 2% of xylan (SERVA, Germany) as the only carbon source. 20 ml of the medium was inoculated with 100 µl of overnight pre-cultured bacteria of the *Lactobacillus buchneri* M B/00077 strain in standard MRS medium. After 1, 2, and 3 days of incubation at 30 °C the biomass was separated by centrifugation (10 min, 10 000 g*)* and the supernatants were collected for measuring xylanolytic activity. The assays were based on a quantification of the reducing sugars released from xylan as a result of the extracellular enzyme excreted to the post-cultured medium acting on a 1% xylan solution prepared in a citrate buffer (pH = 4.8) for 60 min at 35 °C. The reducing sugars released from xylan were determined using the Somogyi-Nelson method as described previously by Ghose and Bisaria^47^. The photometric absorption was determined at 620 nm using a DU 800 UV/VIS spectrophotometer (Beckman Coulter, Inc.). The xylose standard curve was constructed by preparing dilutions of xylose in demineralized water and determining the absorbance of those dilutions at 620 nm after the Somogyi-Nelson reaction.

Xylanolytic activity was expressed as units of enzyme activity per ml (U/ml) and defined as the conversion of 1 µmol of xylose per minute under given assay conditions (35 °C, pH = 4.8).

Additionally the viscosity of the MRS medium with xylan as the only carbon source before inoculation with *Lactobacillus buchneri* M B/00077 and viscosity after 1, 2 and 3 days of incubation with bacteria was measured using a Brookfield viscometer (model DV III Rheometer connected with a TC-100 water bath). The measurements were carried out at 30 °C and spindle SC4-18 with a speed of 10 rpm.

### Silage preparation

The grass of *Spartina pectinata* L. was obtained by collecting energy crops belonging to the Department of Agriculture and Biology of Warsaw University of Life Sciences. The grass was harvested at the end of June, cut into 1 cm pieces and ensiled. Each portion of 10 kg of chopped material was tightly compacted in a plastic barrel (packing density of 230 kg dry matter per m^3^). During compaction the biomass was inoculated with the *Lactobacillus buchneri* M B/00077 strain. For this purpose the biomass of the strain previously grown in standard MRS medium was freeze-dried using a lyophilizer Christ Ralpha 1–4 (Martin Christ Gefriertrocknungsanlagen GmbH, Germany), so the preparation obtained was in the form of a water soluble powder. The number of bacteria in the preparation was log 11.2 CFU/g. 10 g of the preparation was dissolved in 200 ml of sterile demineralized water and sprayed onto the biomass during compaction in a barrel. The calculated number of bacteria added to the biomass was log 8.2 CFU/g of biomass/fresh materials. Silages using a commercial enzyme preparation were also prepared. 15 ml of a xylanase preparation with activity of 8000 U/ml (Xylanase 8000 L, Danisco) was dissolved in 200 ml of sterile demineralized water and sprayed onto the plant material during compaction in a barrel, so that calculated enzyme activity was 0.05 U/g of biomass. Control silages were also prepared without bacteria or the addition of enzymes, but with 200 ml of sterile demineralized water. Each variant of the silage was prepared in three repetitions. The grass compression barrels were hermetically sealed with lids equipped with valves for releasing gas. The barrels were stored at room temperature (between 20 and 23 °C) for three months. After a defined number of days, the barrels were opened. The chemical composition of the ensiled material was determined, as well as Biochemical Methane Potential (BMP) tests.

### Analytical methods

In fresh (after harvest) and ensiled grass, the dry matter (DM) and organic dry matter (ODM) was determined. The DM was determined by drying a sample of the material (25 g) at 105 °C to the constant weight of the sample according to standard S358.2 ASABE. The DM of silages was corrected for the loss of volatiles, as described by Porter and Murray^48^. The organic dry matter (ODM) was determined by burning previously dried samples (1 g) at 550 °C for five hours in a furnace (CARBOLITE RWF 1200). Before measuring the pH-value of silages, 10 g of the sample was homogenized with 100 ml of distilled water for 25 min. Water soluble carbohydrates (WSC) in the ethanol extracts of silages were determined using the Luff-Schoorl method according to the standard PN-R-64784:1994. For further analysis, air dry subsamples of the fresh and ensiled plant material were ground in a cutting mill and sieved using a 1-mm sieve. Crude fat in the silages was determined using the Soxhlet method with the use of a Tecator Soxtec System HT equipped with a 1043 Extractor Unit (FOSS, Denmark). To determine the total protein, neutral-detergent fibre (NDF), acid-detergent fibre (ADF) and acid-detergent lignin (ADL), particular standards were used: PN-EN 13342; PN-EN ISO 16472:2007P and PN-EN ISO 13906:2009P respectively. Fibres were determined using a Fibertec 8000 system (FOSS, Denmark). Cellulose was determined as the difference between the content of ADF and ADL fibre, hemicellulose as the difference between NDF and ADF fibres. ADL fibres were recognized as lignin content.

The HPLC method was used to determine the organic acids in silages and culture broths. Organic acids (lactic, acetic, propionic, and butyric) in silages were extracted from the silage sample for 30 min. The plant material was then separated and the extracts obtained were deproteinized. Water extracts from the silages, as well as an aliquot of the culture broth (5 ml, diluted at 1:2) were mixed with 2.5 ml of Carrez I solution and 2.5 ml of Carrez II solution. The mixtures were centrifuged for 3 min. at 150 rpm. The supernatants were filtered through a 0.45 µm filter (Membrane Solutions, USA) prior to HPLC analysis. The analysis were performed using a Gilson system (Meddleton, USA) equipped with an autosampler (GX-271), a multi-solvent pump (322), a column oven, and RI detector (LDC Analytical, Florida, USA). The organic acids were separated using an Aminex 87HP column (300 mm × 7.8 mm, Bio-Rad Laboratories, Canada, USA) operated at 64 °C. Elution was carried out in isocratic mode with sulfuric acid (pH 3.4) as the mobile phase, at a flow rate of 0.8 ml/min. The quantification was based on a refractive index detector and performed with external standards in duplicate. The results were expressed as mean values. All chemicals used in the study were from Sigma-Aldrich. The chemicals were all of analytical grade.

The digestibility of dry matter (DDM) was calculated as presented by Kim et al.^49^:

1 $$ DDM = 88.9 - 0.779 \times ADF $$

### BMP tests

Anaerobic digestions (AD) of the silages obtained were conducted in glass fermenters (1.3 L) into which 5 g of the material and 100 ml of inoculum (the source of methanogen bacteria) was introduced. The inoculum (the content of secondary digester from an agricultural biogas plant in Poland) was pre-incubated for five days at 39 °C to minimalize the production of biogas from itself. Fermenters were closed with OxiTop Control heads (WTW, Germany) equipped with manometry sensors. Before starting the fermentation process, the fermenters were flushed with N_2_ to create anaerobic conditions. The control assays were fermenters with inoculum without the addition of plant material. The AD was conducted at 39 °C. The fermenters were placed on mixing platforms (WTW, Germany) to guarantee constant mixing of the fermentation mass. Anaerobic digestion lasted until the *plateau* was achieved (until there was no increase in pressure). All assays were prepared in five repetitions.

The pressure of biogas generated inside the fermenters was monitored and saved using OxiTop heads (360 measurements during a defined period of AD), the data were transferred to the OxiTop OC 110 Controller and then to a PC. The data obtained were further processed using the Excel program. The biogas composition was determined using a gas analyser (COMBIMASS GA-m, Germany), which was connected to the side tubes of the fermenters at the end of the fermentation process.

To calculate the volume of biogas obtained with reference to normal conditions (V_o_) the following calculation was used:

2 $$ V_{o}  = p_{1} V_{1} T_{o} /T_{1} p_{o} $$

where *p*_*o*_ is the pressure 1013 hPa; *T*_*o*_ is the temperature of 273 K; *p*_1_ is the biogas pressure (hPa); *V*_1_ is the volume of the glass fermenter (m^3^); *T*_1_ is the temperature of the fermentation process (K).

From the volume of biogas obtained, the volume of biogas produced from the inoculum itself was subtracted, as well as the volume of water vapour, which in given conditions occupies 6.5% of the volume of biogas. Methane yields were calculated as the cumulative volume achieved with reference to the organic dry matter of plant material subjected to anaerobic digestion.

The theoretical (max) production of biogas was calculated based on the chemical composition of the silages, taking into account that from carbohydrates (as the sum of WSC and NDF fibres), fat and protein 800, 1250 and 700 dm^3^/kg_ODM_ of biogas can be obtained respectively^50^. In the calculation, the digestibility of particular constituents was considered as 100%, which means that, theoretically all organic dry matter was converted into biogas.

### Identification of genes encoding endo-1,4-β-xylanase and β-xylosidase

The *Lactobacillus buchneri* M strain was cultured in MRS medium at 30 °C for 1 day. Bacterial chromosomal DNA was then purified using a Genomic Mini Kit (A&A Biotechnology), following the manufacturer’s instructions. Genes encoding endo-1,4-β-xylanase (LBUCD034_0572, EC 3.2.1.8) and β-xylosidase (LBUCD034_0426, EC 3.2.1.37) were investigated (Heinl et al. 2012). Those genes were amplified using the following starters: fr (5′-ATTTGGCAGCCAAGTTAGCGTA) and rev (5′-GAACAGAAGACAGCCCATC) for endo-1,4-β-xylanase; fr (5′-CAATCTCATTGCGGGTGAGGT) and rev (5′-TCAATCAGGCTCCAAATCGTCAA) for β-xylosidase. The primers were designed based on the genes found in the *Lactobacillus buchneri* CD034 strain by Heinl et al.^24^. All PCR reactions were performed using a Pequstar 2 × Gradient thermocycler (Pequlab). PCR reactions were performed in a total volume of 25 µl containing 2 µl of template DNA, 1 µL of each of the primers, 12.5 µl of DraemTaq (ThermoScientific), 8.5 µl of water. The conditions for PCR reactions were as follows: initial denaturation at 95 °C for 2 min, 35 cycles of denaturation at 95 °C for 30 s, annealing at 53 ± 4 °C for 35 s, elongation 72 °C for 1 min and final elongation at 72 °C for 4 min. The products of PCR reaction were analyzed on 1.5% agarose gel. The DNA samples obtained were sequenced using the same primers. The sequences were compared to the GenBank sequences data base using BLAST tools.

### Statistical analysis

The experiments assumed a complete plan with the mean and SEM as the measure of central tendency. One‐factor‐at‐a‐time (OFAT) approach was used to choose the dynamic variables. All experiment were performed in triplicates. One-way analysis of variance (ANOVA) followed by the Tukey post hoc test were used to compare the treatments (*p* < 0.05), Statistica ver. 8.0 software (Statsoft, USA). The compatibility of variable distribution with normal distribution was verified with the Shapiro–Wilk test, while the hypothesis about homogeneity of variance was tested with the Levene test and Brown–Forsythe test.

## Supplementary Information

Supplementary Figure S1.

## Data Availability

The data that support the findings of this study are available from the corresponding author upon reasonable request.
