# Supplementary material for: Silage quality and biogas production from Spartina pectinata L. fermented with a novel xylan-degrading strain of Lactobacillus buchneri M B/00077
Source: Sci Rep. 2021 Jun 23;11:13175. doi: 10.1038/s41598-021-92686-y (PMC8222392; doi:10.1038/s41598-021-92686-y)
Supplement: Supplementary file 1 — Supplementary Figure S1. [file 41598_2021_92686_MOESM1_ESM.docx]

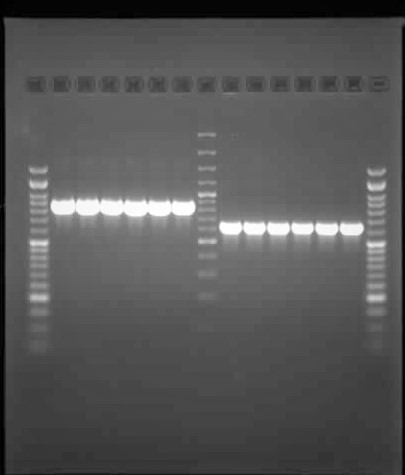


1 2 3 4 5 6 7 8 9 10 11 12 13 14 15

5

6

7

8

9

10

Supplementary Figure 1. Products of PCR reaction in agarose gel. Lane 1, 15 – 50 bp molecule marker; lane 8 – 100 bp molecule marker; 2-7 – products of amplification with endo-1,4-xylanase starters, 9-14- products of amplification with ꞵ-xylosidase starters
